# Incomplete Carney Triad

**DOI:** 10.1210/jcemcr/luae016

**Published:** 2024-02-23

**Authors:** Steven G Waguespack, Neeta Somaiah, Jeffrey E Lee, Khaled M Elsayes

**Affiliations:** Department of Endocrine Neoplasia and Hormonal Disorders, The University of Texas MD Anderson Cancer Center, Houston, TX 77030, USA; Department of Sarcoma Medical Oncology, The University of Texas MD Anderson Cancer Center, Houston, TX 77030, USA; Department of Surgical Oncology, The University of Texas MD Anderson Cancer Center, Houston, TX 77030, USA; Department of Abdominal Imaging, The University of Texas MD Anderson Cancer Center, Houston, TX 77030, USA

**Keywords:** gastrointestinal stromal tumor (GIST), paraganglioma, pulmonary chondroma, succinate dehydrogenase (SDH), *SDHC*

## Image Legend

**Figure 1.**
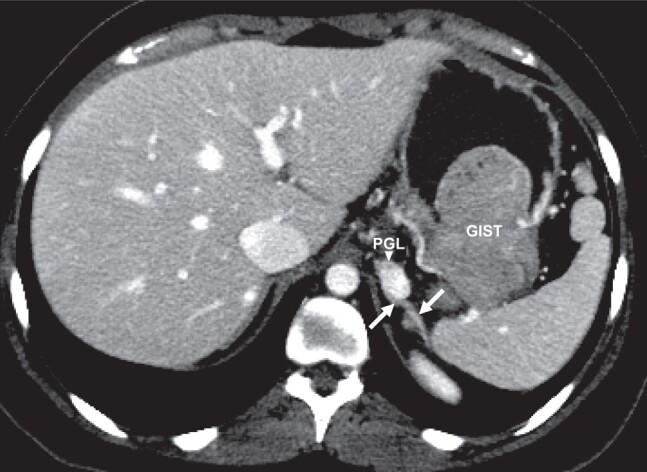
Incomplete Carney Triad in a 20-year-old woman presenting with hematemesis and diagnosed with metastatic gastrointestinal stromal tumor (GIST). An axial image from a contrast-enhanced computed tomography of the abdomen shows a GIST and a paraganglioma (PGL) located adjacent to the normal left adrenal gland (arrows).

A 20-year-old woman with a noncontributory family history was diagnosed with a gastrointestinal stromal tumor (GIST) metastatic to liver and lymph nodes after she developed hematemesis. Computed tomography (Fig. 1) identified multifocal gastric tumors and an intensely enhancing mass (arrowhead) located adjacent to the left adrenal gland (arrows); no lesions were identified in the lungs. The GIST was wild-type (no somatic *KIT* or *PDGFRA* pathogenic variants) and equivocal for succinate dehydrogenase complex, subunit B (SDHB), expression on immunohistochemistry. The patient was treated with imatinib followed by surgery. Because of intense uptake in the left adrenal/periadrenal mass identified on a subsequent ^18^F-fluorodeoxyglucose positron emission tomography/computed tomography scan, a laparoscopic retroperitoneoscopic adrenalectomy was pursued. Preoperatively, she was normotensive with no symptoms of catecholamine excess; fractionated urinary catecholamines and plasma metanephrines were normal. Pathology revealed a 2-cm SDHB-deficient paraganglioma (PGL). Genetic testing revealed no germline pathogenic variants in the genes of the SDH complex or in any other PGL susceptibility gene. The Carney triad is a syndromic but nonhereditary disorder related to *SDHC* promoter hypermethylation (1). It affects young women and is characterized by wild-type, SDH-deficient GISTs, PGLs, and pulmonary chondromas, although not every patient manifests all 3 clinical components, in which case it is known as incomplete Carney triad (2).
